# Association between levels of serotonin, melatonin, cortisol and the clinical condition of patients with rheumatoid arthritis

**DOI:** 10.1007/s00296-023-05296-4

**Published:** 2023-03-13

**Authors:** Aldona Wróbel, Joanna Szklarczyk, Ilona Barańska, Anna Majda, Jolanta Jaworek

**Affiliations:** 1 Laboratory of Nursing Theory and Fundamentals, Institute of Nursing and Midwifey, Faculty of Health Sciences, Jagiellonian University Medical College, Krakow, Poland; 2 Department of Medical Physiology, Institute of Physiotherapy, Faculty of Health Sciences, Jagiellonian University Medical College, Krakow, Poland; 3 Laboratory for Research on Aging Society, Department of Sociology of Medicine, The Chair of Epidemiology and Preventive Medicine, Jagiellonian University Medical College, Krakow, Poland

**Keywords:** Rheumatoid arthritis, Serotonin, Melatonin, Cortisol, Active disease

## Abstract

Rheumatoid arthritis (RA) is a chronic systemic connective tissue disease of autoimmune basis. It is characterized by inflammation of joints and systemic complications. The etiopathogenesis is still unknown. Predisposing factors for the disease include genetic, immunological and environmental. Chronic disease and the stress experienced by patients disrupt the body’s homeostatic state and weaken the human immune system. Reduced immunity and endocrine disruption may influence the development of autoimmune diseases and exacerbate their course. The aim of the study was to investigate whether there is a relationship between the blood levels of hormones such as cortisol, serotonin, melatonin and the clinical status of RA patients as determined by the DAS28 index and CRP protein. A total of 165 people participated in the study of these 84 subjects had RA and the rest were the control group. All participants completed a questionnaire and had their blood drawn to determine hormones. Patients with RA had higher plasma cortisol (324.6 ng/ml vs. 292.9 ng/ml) and serotonin concentrations (67.9 ng/ml vs. 22.1 ng/ml) and lower plasma melatonin (116.8 pg/ml vs. 330.2 pg/ml) compared to controls. Patients whose CRP concentration were above normal also had elevated plasma cortisol concentration. No significant association was observed in RA patients between plasma melatonin, serotonin and DAS28 values. However, it can be concluded that those with high disease activity had lower melatonin levels as compared to patients with low and moderate DAS28 values. Significant differences were found between RA patients not using steroids and plasma cortisol (*p* = 0.035). In RA patients, it was observed that as plasma cortisol concentration increased, the chance of having an elevated DAS28 score, indicating high disease activity, increased.

## Introduction

Rheumatoid arthritis (RA) is a chronic systemic connective tissue disease of autoimmune origin. It is characterized by inflammation of symmetric joints, the occurrence of extraarticular lesions, and systemic complications. The etiopathogenesis of RA is still not fully understood. Many factors are involved in the onset and development of the disease, including genetic, immunological, and environmental factors [1–6]. The chronic inflammatory process in the joints leads to a significant reduction in joints mobility and deformity, contributing to the patient's disability [7–9]. Currently, the 2010 European League Against Rheumatism/American College of Rheumatology (EULAR/ACR) classification criteria are used in the diagnosis of RA. These place emphasis on the diagnosis of the early phase of the RA and the selection of patients with an acute course of the disease with poor prognosis [10–12]. To assess disease activity, the Disease Activity Score 28 (DAS 28) is used most frequently in RA patients [13, 14]. The disease is characterized by alternating periods of exacerbation and remission. Joint destruent depends on the form of RA (seronegative/seropositive), the type of treatment used and the body’s response to therapy.

[4, 7, 8]. Approximately 400,000 people in Poland suffer from RA. Women are affected three times more often than men [15].

The experience of chronic disease is one of the stressors that accompany human life. However, not only can the disease cause stress, but stress can also contribute to the onset of disease [16–19]. Nervous and immune systems interact and communicate through the sympathetic nervous system-adrenal medulla axis and the hypothalamic–pituitary–adrenal cortex axis. Long-term stress disrupts the body’s homeostasis which leads to overload and the occurrence of systemic disorders. Reduced immunity due to a chronic stressful situation, as well as endocrine disorders related to abnormal hormone secretion, can contribute to the development of diseases and exacerbate their course [20, 21]. In this cross-sectional study, we aimed to investigate whether there is a relationship between the levels of hormones involved in stressful situations such as cortisol, serotonin, melatonin and the clinical status of RA patients as determined by the DAS28 index and CRP protein.

## Methods

### Study design

The study was carried out from May 2017 to June 2018 in the Department of Rheumatology and in an outpatient hospital clinic. The approval of the Bioethics Committee was obtained: opinion no. 122.6120.293.2016, date: 28/04/2017, approving institution: Jagiellonian University). The study complies with the Declaration of Helsinki. Written informed consent was obtained from all patients prior to the study's start. All participants in the study completed a questionnaire on disease prevalence, sociodemographic data and rheumatoid arthritis. A blood sample, collected during routine examination of patients, was used to determine hormones. Blood was collected into vacuum test tubes in a volume of 5 ml from the ulnar vein. Between 7.45 and 8.15 a.m., venous blood was drawn to determine plasma level of cortisol, serotonin and melatonin. All assays were performed by immunoenzymatic ELISA. Information on C-reactive protein (CRP) levels and DAS28 index were obtained from medical records, which were determined on the day of the study. Inclusion criteria for the study group were a diagnosis of RA, age between 18 and 70 years, the absence of psychiatric disorders, cancer, metabolic endocrine diseases and patient consent to participate in the study. The control group consisted of patients without a diagnosis of RA and psychiatric disorders, cancer and endocrine diseases, who had no joint pain or swelling on the day of the study, who were between 18 and 70 years old and who consented to participate in the study.

### Statistics

Mean, standard deviation, median, minimum and maximum values were used to describe quantitative variables (hormone concentrations, blood CRP protein, and DAS 28 index). The conformity of the distributions of these variables to a normal distribution was tested using the Shapiro–Wilk test. The control and study groups were compared using the Student’s *t*-test or the Mann–Whitney *U* test. Cross tabulations were used to describe qualitative variables. Differences between the control and study groups were assessed using the *χ*2 test or Fisher’s exact test. Univariable analyses used the *χ*2 test or Fisher’s exact test, Student’s *t*-test or Mann–Whitney *U* test depending on the nature of the variables. Multivariable logistic regression analyses were presented by odds ratio (OR) and 95% confidence intervals (CI). The analyses were carried out using SPSS ver.25. The accepted level of statistical significance was 0.05.

## Result

### Characteristics of the participants

A total of 165 people participated in the study. Of these, 84 people had suffered from RA. The majority were women (78.2%) and people 40 years and older (87.9%). The distribution of education among the study group participants and the control group was similar. Almost a third of the participants lived in rural areas. Only significant differences between the study and the control group were observed in employment status. The control group was dominated by economically active people (71.6%), while the largest group in the study group were retirees (53.6%) (Table 1). Among RA patients, those who had the disease for more than 10 years were the largest group (58%). 16% of the respondents had been ill for 1–3 years, while 11 per cent of the patients had been ill for 6–9 years. The remaining 15% of the patients had been ill for less than 1 year. All RA patients remained on treatment, 60% of whom were treated with glucocorticosteroids.

**Table 1:** Sociodemographic characteristics of the study groups (*n* = 165)

	Total group (*n* = 165)	Study group (RA patients) (*n* = 84)	Control group (*n* = 81)	*p*^a^
	*n* (%)	*n* (%)	*n* (%)	
Gender Female Male	129 (78.2) 36 (21.8)	63 (75.0) 21 (25.0)	66 (81.5) 15 (18.5)	0.314
Age [years] 30–39 40–49 50–59 60–70	20 (12.1) 33 (20.0) 50 (30.3) 62 (37.6)	10(11.9) 16 (19.0) 25 (29.8) 33 (39.3)	10 (12.3) 17 (21.0) 25 (30.9) 29 (35.8)	0.972
Education Primary + vocational Secondary Higher	57 (34.6) 63 (38.2) 45 (27.3)	33 (39.3) 29 (34.5) 22 (26.2)	24 (29.6) 34 (42.0) 23 (28.4)	0.409
Place of residence Village City 50 000 inhabitants City of 50–100 000 inhabitants City > 100 000 inhabitants	62 (37.6) 24 (14.5) 15 (9.1) 64 (38.8)	33(39.3) 12 (14.3) 11 (13.1) 28 (33.3)	29 (35.8) 12 (14.8) 4 (4.9) 36 (44.4)	0.215
Employment status Works Does not work Apprentice/student Retired	80 (48.5) 16 (9.7) 5 (3.0) 64 (38.8)	22 (26.2) 16 (19.0) 1 (1.2) 45 (53.6)	58 (71.6) 0 (0.0) 4 (4.9) 19 (23.5)	< 0.001

^a^*p* for a chi2 test or Fisher’s exact test comparing the distribution of variables between the study and control groups

### Hormone concentration control group vs. study group

**Fig. 1:**
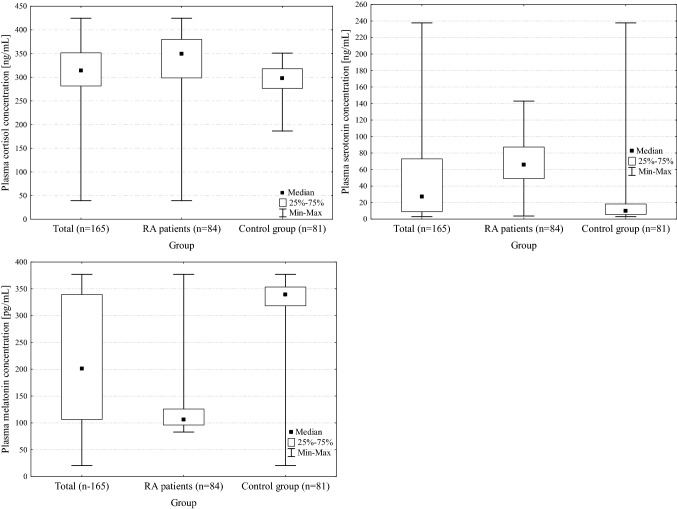
Distribution of cortisol, serotonin and melatonin concentrations among all subjects and by study (RA) and control group

In all subjects, the mean cortisol concentration was 308.9 ng/ml, serotonin 44.2 ng/ml and melatonin 221.6 pg/ml. There were differences in hormone concentrations between the study group and the control group (Fig. 1).

- The mean cortisol concentration was significantly higher (*p* = 0.01) in RA patients (324.6 ng/ml) compared to the control group (292.9 ng/ml);
- The mean serotonin concentration was significantly higher (*p*<0.001) in RA patients (67.9 ng/ml) compared to the control group (22.1 ng/ml);
- The mean melatonin concentration was significantly lower (*p*<0.001) in RA patients (116.8 pg/ml) compared to the control group (330.2 pg/ml).

### Hormones and clinical status of RA patients

The mean concentration of CRP protein in RA patients was 10.26 ± 14.13 mg/l. The results ranged from 0.10 to 76.20 mg/l. In almost half of the patients (46.4%), the CRP value was within the normal range (0.1—5.0 mg/l). In the remaining 53.3% of the patients, the CRP value was above normal. The mean value of DAS28 in RA patients was 3.8 ± 1.24, with results ranging from 1.24 to 6.8. The largest group (50.0%) had low disease activity, followed by those with moderate activity (27.4%), and the smallest percentage of patients showed high activity (21.4%). Of all subjects, only one was in remission of the disease.

No statistically significant correlation was found between melatonin concentration and plasma CRP protein concentrations in RA patients. The median concentrations of melatonin in both groups were very similar and were 116.0 ± 26.0 pg/ml and 117.3 ± 47.7 pg/ml in the normal CRP and in patients with elevated CRP protein, respectively. Taking into account the median value, it can be observed that in all subjects in both groups, the median concentrations of melatonin were higher in those whose CRP protein concentration was within normal limits (up to 5 pg/ml). The analysis performed in the gender groups also showed that melatonin values were higher among people with normal CRP. There was also no relationship between plasma serotonin concentration and CRP protein concentration. However, it can be concluded that, considering both the study group (women and men combined) and the men's group alone, plasma serotonin concentrations were higher in those respondents with normal CRP values.

When analyzing the distributions of plasma cortisol concentrations in total for all subjects in the study group, as well as considering the female and male groups separately in relation to CRP values, it was observed that patients with elevated CRP also had higher plasma cortisol concentrations.

When analyzing cortisol concentrations in RA patients who did not receiving steroid therapy, it was observed that in the whole group, as well as separately in the women's group and separately in the men’s group, cortisol concentrations were significantly higher in those with elevated serum CRP values (Table 2).

**Table 2:** Relationship between cortisol concentration and plasma CRP protein concentration among RA patients without glucocorticosteroid treatment (*n* = 34)

CRP Concentration^b^	Cortisol concetration [ng/ml]		
	RA patients	Women	Men
Normal (5 mg/l)	(*n* = 18)	(*n* = 14)	(*n* = 4)
Mean (SD)	336.3 (39.5)	336.7 (43.5)	334.9 (24.8)
Median	330.1	330.1	338.3
Min—Max	268.1–416.5	268.1–416.5	304.7–358.6
Elevated (> 5 mg/l)	(*n* = 16)	(*n* = 8)	(*n* = 8)
Mean (SD)	374.9 (27.4)	380.2 (30.01)	369.8 (25.3)
Median	381.4	384.9	378.4
Min—Max	331.1–421.6	343.3–421.6	331.1–398.2
p^a^	0.003	0.024	0.048

^a^p for the Mann–Whitney *U* test to assess the association between CRP protein concentration and cortisol concentration

^b^C-reactive protein

No statistically significant association was observed between plasma melatonin concentration in RA patients and the value of the DAS28 index. However, it can be concluded that those with moderate or low disease activity had slightly higher concentration of melatonin compared to those with high disease activity (117.8 pg/ml vs 113.2 pg/ml; *p* = 0.394).

When analyzing the relationship between serotonin concentration and DAS28 values, it was observed that for the entire study group, plasma serotonin levels was similar in all patients groups with low and moderate and high disease activity (68.0 pg/ml vs 67.3 pg/ml, respectively; *p* = 0.889). Women with low and moderate disease activity had lower levels of serotonin compared to women with high disease activity (68.9 pg/ml vs 74.4 pg/ml; *p* = 0.658). In men, an inverse relationship was observed (63.7 pg/ml vs 56.6 pg/ml; *p* = 0.368).

There was no statistically significant relationship between the value of DAS28 and plasma cortisol concentration. However, it was observed that, among RA patients, those with low or moderate disease activity presented lower cortisol concentration compared to those with high disease activity (310.7 ± 97.7 ng/ml vs. 356.8 ± 48.2 ng/ml, respectively; *p* = 0.080).

Patients with low to moderate disease activity who did not receive steroid therapy had significantly lower plasma cortisol concentration compared to RA patients who had high disease activity (347.3 ± 37.9 ng/ml vs. 379.6 ± 34.3 ng/ml; *p* = 0.035). This relationship was found for the entire study group, as well as for the female group and the male group (Table 3).

**Table 3:** Plasma cortisol concentrations in relation to the value of the DAS28 index among RA patients without glucocorticosteroid treatment (*n* = 34)

DAS28 index^b^	Cortisol concetration [ng/ml]		
	RA patients	Women	Men
Low or moderate activity	(*n* = 27)	(*n* = 19)	(*n* = 8)
Mean (SD)	347.3 (37.9)	346.1 (37.1)	350.3 (27.6)
Median	351.5	407.4	355.0
Min—Max	268.1–416.5	351.5–421.6	304.7–382.9
High activity	(*n* = 7)	(*n* = 3)	(*n* = 4)
Mean (SD)	379.6 (34.3)	393.5 (37.1)	365.7 (31.3)
Median	382.9	407.4	373.8
Min—Max	331.1–421.6	351.5–421.6	331.1–392.1
p^a^	0.035	0.087	0.214

^a^p for the Mann–Whitney *U* test to assess the association between DAS28 and cortisol concentration

^b^Disease activity score

### Multivariable analyses

Sex, age, place of residence and plasma serotonin and cortisol were not significantly associated with the chance of developing RA disease. However, it was observed that as blood melatonin concentrations increased, the chance of developing the disease decreased (OR = 0.95, 95% CI 0.93–0.98) (Table 4).

**Table 4:** Multivariable logistic regression analysis of the association of selected characteristics with the chance of occurrence of RA (*n* = 165)

Dependent variable: Occurrence of RA (yes vs. no)	OR^a^	95% CI^b^	*p*
Gender			
Female	1.00		
Male	0.12	0.01–2.85	0.188
Age			
30–49	1.00		
50–59	0.23	0.004–12.5	0.471
60–70	0.24	0.01–11.0	0.467
City of residence			
Rural	1.00		
City	1.25	0.07–23.20	0.880
Melatonin [pg/mL]	0.95	0.93–0.98	< 0.001
Serotonin [ng/mL]	1.02	0.99–1.05	0.184
Cortisol [ng/mL]	0.99	0.97–1.02	0.858

^a^odds ratio

^b^95% confidence interval

The next study model presented data on the relationship between high CRP and sex, age, and plasma concentrations of the hormones studied: melatonin, serotonin, and cortisol. No statistically significant association was observed, but it was found that men and those with high stress were more likely to have elevated CRP protein concentrations (Table 5).

**Table 5:** Multivariable logistic regression analysis of the association of selected characteristics with elevated plasma CRP protein concentrations and high disease activity (DAS 28) (*n* = 84)

	CRP^a^ (elevated vs. normal)			DAS28 index^b^ (high activity vs. low or moderate)		
	OR^c^	95%CI^d^	*p*	OR^a^	95%CI^b^	*p*
Gender						
Female	1.00			1.00		
Male	2.63	0.69–10.02	0.156	4.53	0.96–21.34	0.056
Age						
18–49	1.00			1.00		
50–59	0.92	0.23–3.66	0.903	1.47	0.24–8.98	0.677
60–70	1.93	0.55–6.75	0.304	0.84	0.16–4.32	0.833
Melatonin [pg/ml]	1.01	0.98–1.03	0.607	1.02	0.99–1.05	0.210
Serotonina[ng/ml]	0.99	0.98–1.01	0.756	0.99	0.97–1.02	0.853
Cortisol [ng/ml]	1.00	0.99–1.01	0.165	1.02	1.0–1.03	0.05

^a^C-reactive protein

^b^Disease activity score

^c^Odds ratio

^d^95% confidence interval

In multivariable analyses, men, compared to women, were found to have more than four times the chance of high disease activity (OR = 4.53, 95% CI 0.96–21.34). In RA patients, it was observed that as plasma cortisol concentration increased, the chance of having an elevated DAS28 score, indicating high disease activity, increased (Table 5).

## Discussion

Stress can mobilize the body to fight, but it can also contribute to weakening it. The very occurrence of a chronic disease becomes a source of severe stress. Stress can be said to influence not only the onset of the disease, but also the health status of the patient [22–24].

Depending on its type, duration and intensity, stress inhibits or stimulates the immune response. The strain on the body caused by an overreaction to a stressful stimulus activates the nervous system, the endocrine system and the immune system. These systems interact with each other so that the body can adapt to the resulting stress situation. In the event of an overload or stressful situation, endocrine disorders occur, among other things. These disorders can negatively affect health, especially in people with immune dysfunctions, such as RA patients [17, 20–22].

In our study, plasma concentrations of hormones such as serotonin, melatonin, and cortisol were determined. There were significant differences in these hormone concentrations between the RA group and the control group. Mean cortisol and serotonin were significantly higher among patients with RA compared to the people from the control group. On the other hand, mean melatonin concentrations were significantly lower in RA patients compared to the non-RA subjected.

Paolino et al*.* reported differences in cortisol and melatonin levels in RA patients compared to healthy subjects. These investigators focus on the morning symptoms of RA (morning stiffness, joint pain, functional disability) associated with a diurnal increase in nocturnal inflammation caused by inadequate cortisol and melatonin secretion in the blood of patients [25]. Researchers Straub and Cutolo also described differences in cortisol concentration in RA patients with active phase of disease. In these patients was observed elevated blood cortisol levels compared to healthy subjects. This was related to inhibition of inflammation during the day versus its regulation in the evening and at night, resulting in increased inflammation in the early morning hours. In addition to cortisol, they also reported on the diurnal rhythm of melatonin, but here they did not show that the concentration of this hormone differed significantly in RA patients compared to healthy controls [26].

The clinical status of RA patients was determined by the disease activity score (DAS 28) and the levels of C-reactive proteins (CRP). Half of the subjects (50%) presented low disease activity. Above-normal CRP protein levels were present in 53.3% of RA patients Bernardes et al. drew attention to the increasing number of studies in which RA patients were characterized by bone loss and increased levels of serotonin in the blood. Increased concentration of serotonin in the plasma of patients with RA compared to people from the control group was also noted in our own research [27].

When examining the relationship between the CRP protein and hormone concentrations, it was found that there was no relationship between the serotonin and melatonin plasma concentrations and the value of the CRP protein in RA patients. In contrast, it could be observed that RA patients (both glucocorticoid treated and non-corticosteroid treated) who had elevated CRP also had higher serum cortisol concentrations.

When looking for an association between the DAS 28 index and hormone concentrations, no significant statistical association was found. However, it can be observed that patients with moderate and low disease activity had slightly higher melatonin concentrations compared to patients with high RA activity. Serotonin and cortisol levels tested were higher in RA patients with high disease activity compared to patients with moderate and low DAS28.

There has been increasing interest in the etiopathogenesis of autoimmune diseases in the population due to the increasing prevalence of these diseases in recent years. One of the factors predisposing to autoimmune diseases could be probably related to the abnormal release of melatonin due to disruption of its circadian rhythm. The uncontrolled inflammatory response and deficient defense system of the organism lead to chronic activation of immune cells and the onset of autoimmune diseases. There is an emerging body of research indicating that melatonin inhibits pro-inflammatory Th17 cells, that play a key role in autoimmune diseases and shifts the balance of the immune response toward immunosuppression [28–30]. In relation to RA, the data on the effect of melatonin on disease severity are contradictory. Some studies suggest that melatonin enhances pro-inflammatory effects and, thus, promotes disease activity in RA, while other publications has documented significant anti-inflammatory and immunoregulatory properties of melatonin in preclinical models of arthritis [31, 32]. In our study, we observed that higher plasma levels of melatonin were present in patients with low disease activity. Based on this observation it could be concluded that, with increasing plasma melatonin concentrations, the chance of developing RA decreased significantly. In contrast, sex, age, place of residence, serotonin, and cortisol concentrations were not significantly associated with that chance.

The interaction between stress and hormonal changes in RA patients requires further research. It is important to bear in mind that this interaction may occur at multiple levels, which may influence the occurrence and activity status of autoimmune diseases.

## Strengths and limitations

A limitation of this study was the small study group, making the study unrepresentative. However, it is one of the first studies in Poland to examine melatonin, serotonin and cortisol concentrations and the clinical status of RA patients. A strength of the study was the inclusion of a control group that was comparable and an objective measurement examining hormone levels, inflammation and disease activity.

## Conclusion

The study showed that RA patients had significantly higher cortisol and serotonin levels and lower melatonin levels compared to controls. Multivariate analyses showed that increasing melatonin levels modify the progression of RA and clinical condition. For patients without corticosteroid treatment, a correlation was observed between cortisol levels and CRP levels and DAS28 scores. Patients with elevated CRP and high disease activity scores had higher cortisol levels. These findings highlight the importance of conducting further research to clarify the role of hormones in the development of RA and its course, especially as the data presented by researchers on the role of hormones in RA are contradictory.

## Data Availability

The datasets generated during and/or analysed during the current study are available from the corresponding author on reasonable request.
